# Influence of Pre-Operative Estimation of Draf III Expected Diameter on Surgical Outcome

**DOI:** 10.3390/jpm14090951

**Published:** 2024-09-09

**Authors:** Matteo Alicandri-Ciufelli, Carla Cantaffa, Margherita Basso, Vito Colacurcio, Daniele Marchioni, Daniela Lucidi

**Affiliations:** Department of Otolaryngology Head and Neck Surgery, University Hospital of Modena, 41121 Modena, Italy; matteo.alicandri@unimore.it (M.A.-C.); carla.cantaffa@outlook.com (C.C.);

**Keywords:** DRAF III, endoscopic transnasal approach, modified Lothrop procedure, frontal sinusotomy

## Abstract

**Introduction**: The DRAF III procedure is indicated for the treatment of benign and selected malignant frontal sinus pathology. Several attempts have been made to find an intra or pre-operative measurement that was predictive of the feasibility of this operation and its success. Among those, the frontal sinus outflow tract anteroposterior diameter (FOAP) appears to be the one with the highest applicability in clinical practice, since it is easy to measure on pre-operative CT scan. The objective of the study is to evaluate whether the minimum and maximum frontal sinus outflow anteroposterior diameters (FOAP min and FOAP max) are associated with the risk of failure and consequently with the need for surgical revision. Secondly, we ought to evaluate whether the angle between the glabella and the nasion on the sagittal plane could influence surgical outcome. **Material and Methods**: This is a retrospective study on patients who underwent DRAF III for benign pathologies at a tertiary Italian referral center from January 2000 to July 2022. FOAP min and FOAP max were measured on the mid-sagittal sections of a pre-operative CT scan. The glabella–nasion angle (G-N angle) was calculated on the same sagittal section. These measurements were compared between patients who experienced a recurrence and those who did not. If a post-operative CT scan was available, the obtained anteroposterior diameter (OD) of the frontal sinus neo-ostium was also measured on the same section. A correlation analysis was performed between the three pre-operative radiologic parameters and OD. **Results**: Twenty-nine patients were included in the study. Six patients experienced restenosis requiring surgical revision. The median FOAP max resulted significantly higher in patients who did not experience recurrence (median: 14.8 mm, IQR: 3.84) than in patients who experienced a recurrence (median: 11.9 mm, IQR: 1.14) (*p* = 0.04). The FOAP max also showed a good positive correlation with OD (*p* = 0.0001; r = 0.7). The correlation between FOAP min and OD was not statistically significant, nor was a correlation found between FOAP min and recurrence risk. The G-N angle was not significantly different in patients who experienced recurrence and those who did not, nor did it show a correlation with OD. **Discussion**: The FOAP max might be a valid pre-operative radiologic parameter to guide a surgeon’s approach to a DRAF III procedure, as it is easy to measure, shows a good correlation with OD, and seems to influence the risk of recurrence.

## 1. Introduction

First described in 1991, what is nowadays known as DRAF III, or the endoscopic modified Lothrop procedure, is indicated for the treatment of benign and selected malignant frontal sinus pathology after failure of more conservative approaches, or as a primary surgery in cases with high risk of failure. These include aspirin-exacerbated respiratory disease; cases with high CT score according to Lund–Mackay or Kennedy, despite maximal medical therapy in patients with chronic rinosinusitis with nasal polyps (CRSwNP); eosinophilic CRS; bronchial asthma; analgesics intolerance; and cystic fibrosis or ciliary dyskinesia, and its associated diseases [1,2].

Through a DRAF III frontal sinusotomy, a single large frontal sinus outflow tract is obtained after removal of the superior nasal septum, the frontal intersinus septum, and the nasal floor of the frontal sinus. With the advantages of avoiding external incisions and allowing for easier detection of recurrences, endoscopic approaches are gradually replacing standard open procedures, thanks to advances in endoscopic technology and image guidance. However, endoscopic approaches to the frontal sinus, specifically, are only recently becoming part of this scenario. The reason why they have been long overlooked is because the anatomy of the frontal sinus outflow tract can be extremely complex in some cases, due to the variable degree of pneumatization of adjacent ethmoidal cells, which inevitably influences the direction and position of the frontal recess [3]. Furthermore, endoscopic approaches to the frontal sinus are hindered by its close vicinity to very delicate structures, so that particular care must be taken to correctly identify anatomical landmarks for the cribriform plate [4], the anterior ethmoidal artery, and lamina papyracea. With increased anatomical knowledge and improved pre-operatory imaging techniques, indications to frontal sinus endoscopic approaches have gradually increased; however, the influence of anatomical variants on post-operative outcomes cannot be understated [5]. Many radiological and intraoperative measurements were proposed over the past years to define the perfect candidate for DRAF III. Among those, the frontal sinus outflow tract anteroposterior diameter (FOAP), proposed by Zhang and colleagues, seems, to the authors of this paper, to be the one with the highest applicability in clinical practice, since it is easy to measure on a pre-operative CT scan [6]. Similarly, different authors have proposed a wide variety of methods to determine the patency of the frontal sinus outflow tract after this procedure [7].

It is known that, due to neoosteogenesis and inflammatory mucosal changes, up to 45% of patients subjected to a DRAF III experience a certain degree of restenosis, especially in the first 12 months from surgery, even though the results are very heterogenous among the published series. However, it seems clear that restenosis does not necessarily require reintervention [8,9].

In light of this premise, the aim of our study was to measure the minimum and maximum FOAP on a pre-operative mid-sagittal CT scan in our patients’ cohort, and determine whether this measurement could be associated with the risk of failure, which is not defined here as the rate of restenosis, but as a need for surgical revision.

Secondly, we sought to evaluate whether another pre-operative parameter, the angle between the glabella and the nasion on the sagittal plane, could influence surgical outcome, since in the senior authors’ personal experience, a more acute angle implies more difficult access to the frontal sinus.

## 2. Material and Methods

This is a retrospective study on patients consequently admitted to the ENT Unit, Head and Neck Surgery Department, of Modena University Hospital, a tertiary referral center, from January 2000 to July 2022, who were subjected to a DRAF III procedure.

**Figure 1:**
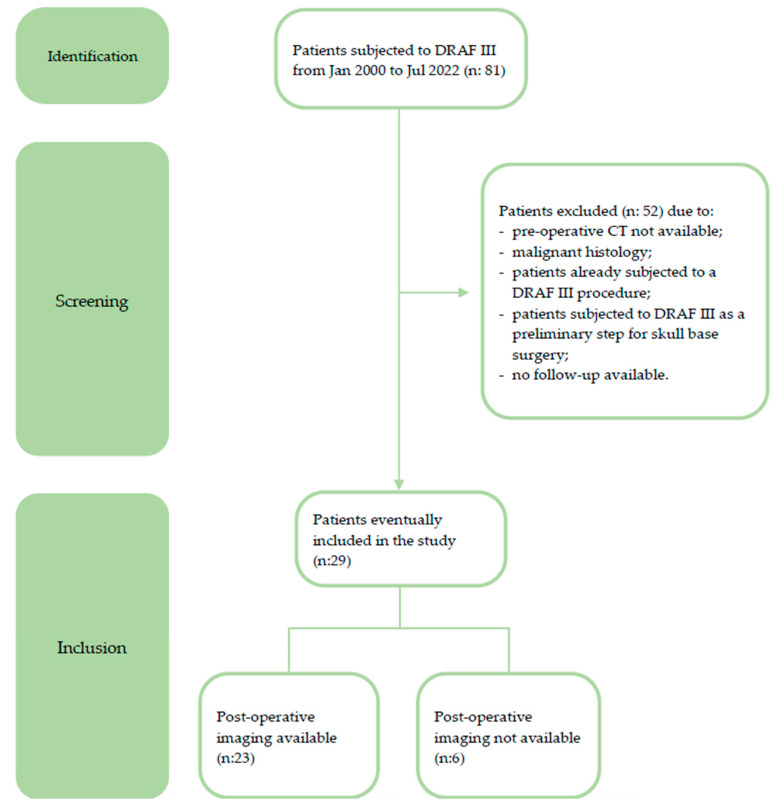
Flowchart of patient selection and data analysis.

The inclusion criteria were the following: patients with benign frontal sinus pathology subjected to a DRAF III procedure, performed both as a primary procedure and after failure of a more conservative endoscopic approach; availability of a pre-operative CT scan; and follow-up time longer than 12 months. Patients with malignant frontal sinus lesions, patients who had already undergone a DRAF III surgery, and patients who were subjected to a DRAF III procedure as a preliminary step for anterior skull base surgery were excluded. The patient selection flowchart is further detailed in Figure 1.

CT scan was the pre-operative imaging modality of choice, since it is widely recognized as the gold standard in pre-endoscopic sinus surgery workup for a number of reasons: its depiction of anatomical features improves surgical planning, it possesses a high sensitivity and specificity when compared with intraoperative findings [10,11], and it allows a good understanding of bony landmarks for neighboring delicate structures. In addition, it is readily available in most centers and less expensive than other imaging modalities.

An early post-operative CT scan (performed during the hospital stay, i.e., 24 to 72 h post-operatively) was prescribed in selected patients, under the surgeon’s request and based on the intraoperative anatomy and need for extensive manipulation.

All patients at our center undergo a uniform post-operative follow-up schedule as follows: follow-up visits begin 1 week post-operatively, then 1 month and 3 months post-operatively, thereafter as needed. Patients who presented with symptoms such as headaches, nasal congestion, and/or endoscopic signs such as frontal neo-ostial mucosal edema or obstruction at the endoscopic examination and patients where endoscopic exploration of the frontal sinus was not feasible were prescribed a post-operative CT scan.

Cases with radiologic evidence of recurrence were then subjected to surgical revision. Patients who experienced recurrence and those who did not were divided into two groups (recurrent patients or RP, and non-recurrent patients or NRP).

The demographic data of the patients, clinical and surgical information were collected from clinical charts and tabularized. An electronic Excel database was filled out with the clinical and pathological characteristics of the enrolled patients.

**Figure 2:**
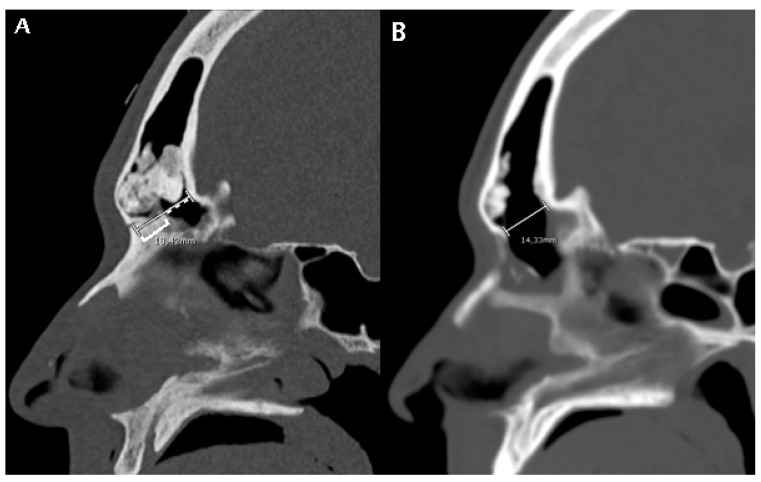
A patient subjected to a DRAF III for relapse of osteoma and fronto-orbital mucocele. (**A**) shows a pre-operative CT scan where FOAP max (white line) is measured as the sum of the mid-sagittal thickness of the nasal beak (white square bracket) and the mid-sagittal distance between the nasal beak and the most anterior skull base (white dotted line). (**B**) shows the post-operative CT scan on the same mid-sagittal section. The white line represents the maximum anteroposterior diameter of the frontal sinus neostium after surgery, which we termed obtained diameter (OD, see Figure 5).

High-resolution CT images were analyzed from all the patients included by two authors (C.C. and M.B.). Mid-sagittal images, identified by the location of the nasal septum and crista galli, were chosen for analysis and measurement. The minimum and maximum frontal sinus outflow anteroposterior diameters (FOAP min and FOAP max, respectively) were measured. The FOAP max was measured as the sum of 1) the mid-sagittal anteroposterior thickness of the nasal beak and 2) the mid-sagittal anteroposterior distance between the nasal beak and the anterior most skull base (Figure 2), as described in the study by Zhang et al. It corresponds to the distance between the nasion and the posterior wall of the frontal sinus.

**Figure 3:**
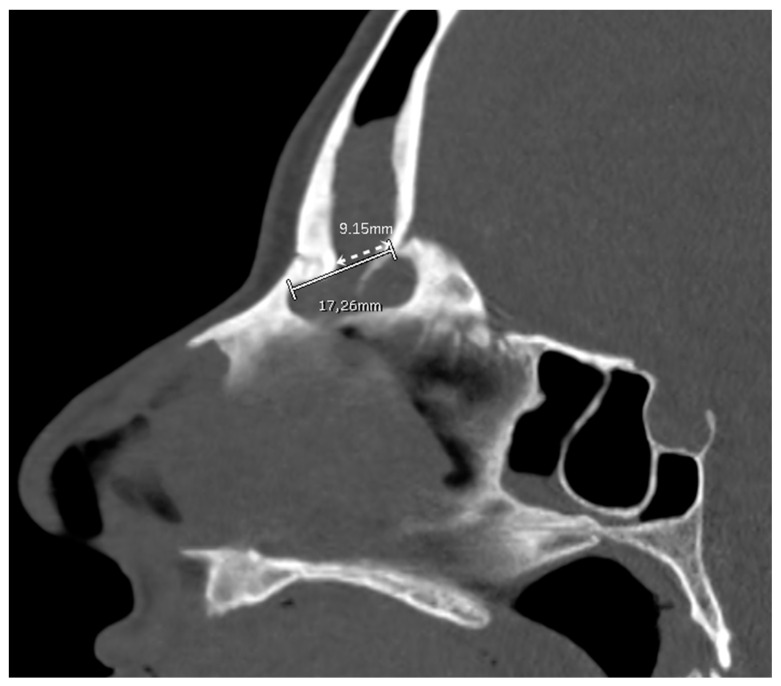
A patient undergoing a DRAF III for bilateral fronto-ethmoidal rhinosinusitis. On the pre-operative sagittal CT scan, the FOAP min (dashed white line with arrows) is measured as the distance between the posterior edge of the frontal beak and the posterior wall of the frontal sinus. The solid white line corresponds to the FOAP max.

The FOAP min was measured as the distance between the posterior edge of the frontal beak and the posterior wall of the frontal sinus, following the indications of the previously mentioned article (Figure 3). 

**Figure 4:**
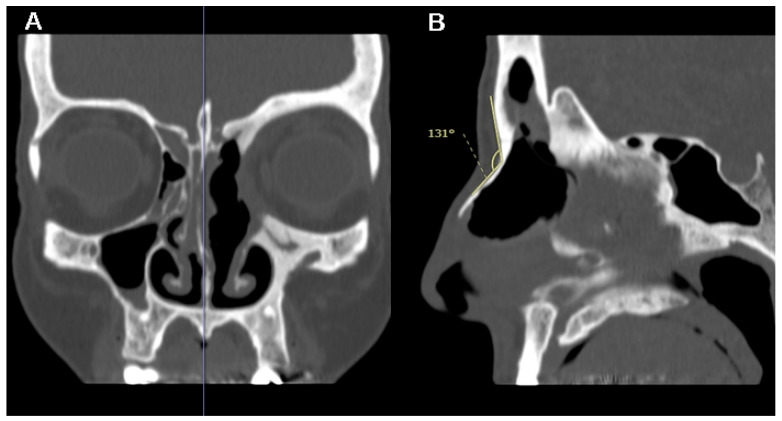
A patient with left fronto-ethmoidal sinusitis with orbital involvement undergoing a DRAF III surgery. On the coronal CT-scan, the mid-sagittal plane is identified by the location of nasal septum and crista galli (blue vertical line in (**A**)); the angle between the glabella and nasion (G-N angle) is then calculated on exactly these mid-sagittal sections (**B**).

The angle between the glabella and nasion (G-N angle) was calculated on the same sagittal section (as shown in Figure 4). Since the G-N angle is not affected by surgery, it can be measured on both pre- and post-operative scans.

The FOAP min, FOAP max and G-N angle were then compared between RP and NRP groups using the Mann–Whitney test for unpaired ranks.

**Figure 5:**
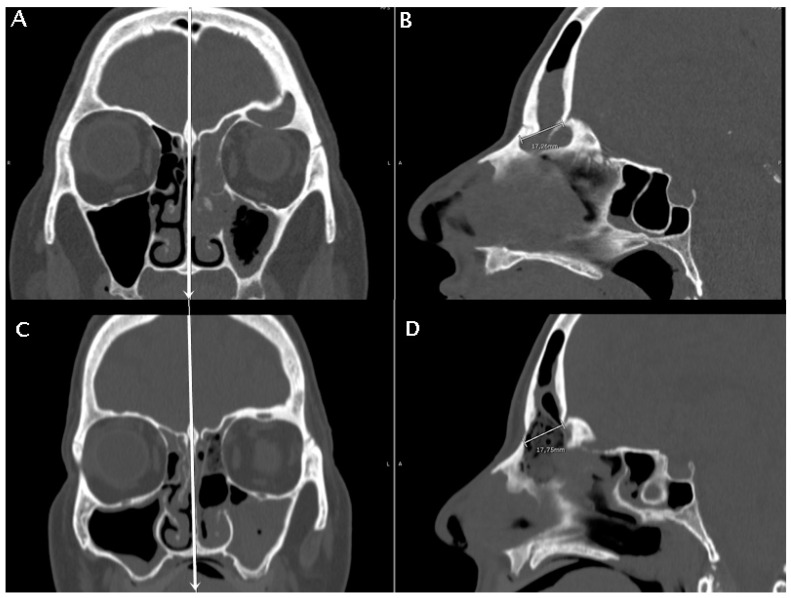
A patient with fronto-ethmoidal inverted papilloma undergoing a DRAF III surgery. The pre-operative CT scan is in (**A**,**B**), the post-operative CT scan is in (**C**,**D**). Coronal sections are used to identify the mid-sagittal line by the location of nasal septum and crista galli (white vertical lines in (**A**,**C**)). The anteroposterior diameter of the frontal sinus ostium is then measured pre- and post-operatively on mid-sagittal sections (**B**,**D**). The anteroposterior diameter of the frontal sinus neo-ostium in the post-operatory mid-sagittal images (**D**) was termed the obtained diameter (OD).

In the subset of patients with available early post-operative CT scan, a post-operative radiological parameter was measured, that is the obtained diameter (OD). It was calculated on the same mid-sagittal section of the pre-operative CT scan, as the distance between the most anterior portion of the frontal neo-ostium and the most anterior portion of the anterior skull base, as shown in Figure 5. A correlation analysis between the FOAP min, FOAP max, and G-N angle with OD was performed by means of the Pearson correlation coefficient for continuous variables.

Statistical analysis was performed by means of Graph Pad Prism software version 7.0 (San Diego, CA, USA). Logistic regression analysis was performed by means of R. Statistical significance was set at a *p*-value < 0.05. The 95% confidence intervals (CI) of each statistical analysis were reported. For the descriptive analysis, the variables were reported as the median and interquartile range, given that the small size of the sample did not allow to assume a normal distribution.

This research was conducted in full accordance with the World Medical Association Declaration of Helsinki (2002). Informed consent was obtained from all participants in written form. For this kind of retrospective investigation, our institutional review board does not perform a formal ethical assessment.

## 3. Results

Twenty-nine patients were enrolled in the study. In total, 11 were females (37.9%), while 18 were males (62.1%). The mean age of enrolled patients was 53.7 years old (range: 18–79 years). The diagnosis was mucocele in 15 cases (51.7%), chronic rhinosinusitis with nasal polyps (CRSwNP) in 3 cases (10.3%), CRSwNP with associated frontal mucocele in 1 case (3.4%), chronic rhinosinusitis without nasal polyps (CRSsNP) in 7 cases (24.1%), osteoma in 1 case (3.4%), inverted papilloma in 1 case (3.4%) and fibrous dysplasia in 1 case (3.4%). The other patients’ characteristics are summarized in Table 1.

In total, 21 out of 29 patients (72.4%) had already received endoscopic sinonasal surgery. Two cases of intraoperative cerebrospinal fluid leak were registered. In both cases, an intraoperative repair was performed and there was a complete resolution during the hospital stay.

The median follow-up time, calculated as the time between surgery and the last endoscopic examination, was 66 months (IQR: 34, range: 16–157 months). In total, 2 patients with mucocele, 1 patient with CRSwNP associated with frontal mucocele, 1 patient with fibrous dysplasia, and 2 patients with CRSsNP experienced restenosis requiring surgical revision, for a total of 6 out of 29 patients (20.7%) after a mean follow-up time of 40 months (IQR: 34; range: 14–80 months).

Four patients underwent a revision DRAF III procedure, whereas one patient was prescribed an osteoplastic flap approach, including sinus obliteration. One patient was referred to biological treatment with monoclonal antibodies. The characteristics of patients with recurrences requiring surgical revision are summarized in Table 2.

All patients had satisfactory mid-sagittal images. The median FOAP max in the entire study cohort was 14 mm (IQR: 4; range: 7.67–19.16 mm). When the RP and NRP groups were compared, the median FOAP max resulted as being significantly higher in patients who did not experience recurrence (median: 14.8 mm, IQR: 3.84) than in patients who experienced a recurrence (median: 11.9 mm, IQR: 1.14) at the Mann–Whitney test (*p* = 0.04). However, no significance was obtained in the univariate logistic regression analysis (*p* = 0.13).

**Figure 6:**
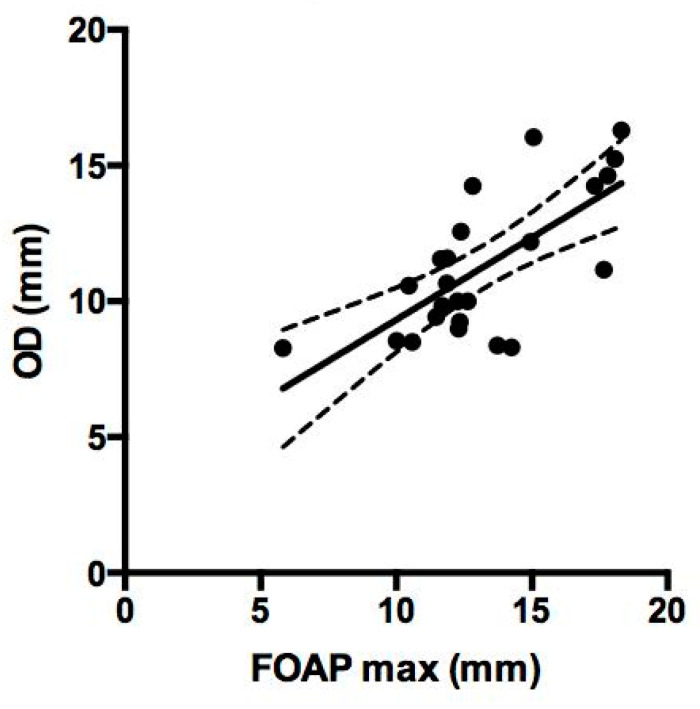
Correlation between FOAP max and obtained frontal sinus anteroposterior diameter (OD) was statistically significant (*p* = 0.0001; r = 0.7).

Early post-operative CT scan was available for all but six patients. The median OD was 12 mm (IQR: 2.06, range: 8.28–16.29 mm). The correlation between the FOAP max and the obtained frontal sinus anteroposterior diameter was statistically significant (*p* = 0.0001; r = 0.7), as shown in Figure 6. However, when the RP and NRP groups were compared, a significant difference in terms of median OD was not obtained (*p* = 0.79), although the median OD was higher in patients who did not experience recurrence (median: 11.35 mm, IQR: 5.26 mm) than in patients who experienced a recurrence (median: 10.96 mm, IQR: 1.67 mm).

The median FOAP min in the entire study cohort was 8 mm (IQR: 3.49 mm, range 4.63–15.08 mm). The correlation between the FOAP min and OD was not statistically significant (*p* = 0.45; r = 0.17). The FOAP min was not significantly different in the NRP and RP groups (*p* = 0.07).

The median G-N angle in the entire study cohort was 129° (IQR: 21.5°, range 101–147°). The correlation between the G-N angle and OD was not statistically significant (*p* = 0.49; r = −0.19). G-N was not significantly different in the NRP and RP groups (*p* = 0.11). The results are summarized in Table 3.

## 4. Discussion

Since the introduction of endoscopic sinus surgery, approaches to the frontal sinus have always been considered as most challenging, due to the anatomical variability of the frontal sinus region [12]. A DRAF III frontal sinusotomy indeed requires the introduction of powered drills in a small area which is bounded by very delicate structures. In addition, the aim of this procedure, when employed as a treatment for benign inflammatory disease, is to guarantee a single large frontal sinus outflow tract to prevent recurrence, hence the importance of accurate patient selection. In fact, a certain degree of restenosis of the frontal sinus outflow tract is to be expected, due to a combination of neoosteogenesis, scarring, and adhesions, especially in patients with persistent inflammation [13]. A narrow frontal ostium also increases the risk of frontal mucosal stripping during surgical maneuvers, thus increasing the probability of iatrogenic scar formation and restenosis [14].

A number of radiologic parameters have been proposed over the past year to assist in the pre-operative selection of patients with frontal sinus pathology. Actually, the role of the CT scan is openly recognized as fundamental in the surgical planning of endoscopic sinus surgery as a whole. For instance, a few recent interesting studies have been published exploring the inter-individual variability of the anterior ethmoidal artery, which can be assessed pre-operatively on CT scan, thus minimizing the risk of intraoperative bleeding [15,16]. A number of other anatomical parameters can be pre-operatively evaluated through CT scans, such as the structure of the lamina papyracea [17]; the anatomy of the olfactory fossa [18]; sphenoid sinus pneumatization patterns, which affect the location of the sphenoid ostium and its relationship to adjacent structures [19,20,21]; and the position of the vidian canal [22], just to name a few.

Regarding endoscopic approaches to the frontal sinus, already in 2001, Gross et al. suggested, based on personal experience, that appropriate candidates for a DRAF III frontal sinusotomy should have a frontal beak thickness not exceeding 1 cm and an anteroposterior diameter at the cephalad margin of the frontal recess of at least 1.5 cm [23]. However, in a cadaveric study by Farhat et al., the authors observed that access to the frontal sinus could be safe even in specimens with an anteroposterior diameter shorter than 1.5 cm, as long as the thickness of the nasal beak was less than 1 cm and the accessible dimension (the distance between two parallel lines lying in the parasagittal plane through the midportion of the internal frontal ostium) was large enough (≥5 mm) [24]. It was later shown that less than half of the population would meet these criteria and thus be eligible for an endoscopic approach [25].

In a study by Gheriani and colleagues, the frontal ostium diameter (FOD) is defined as the distance between a reference line passing through the most prominent projection of the frontal beak and perpendicular to it (R-line) and a second line passing through the deflection point of the anterior skull base (S-line) and parallel to the R-line [26]. They observed that FOD values smaller than 7.5 mm were associated with longer operative times and therefore higher surgical difficulty. The cut-off value of 7.5 mm was obtained by calculating the median FOD of all patients enrolled in the study. However, no data on follow-up are present in this study.

Later, Zhang et al. demonstrated that the pre-operative distance between the nasal beak and the anterior skull base, which they referred to as the minimum frontal ostium anteroposterior diameter (FOAP-min), correlated with the frontal neo-ostium contour area at one year post-operation. A weaker correlation was also observed for the maximum frontal anteroposterior diameter (FOAP-max), which corresponds to the distance between the outer limit of the frontal beak and the inner portion of the anterior skull base, but it lost significance on multivariate analysis. This study was the first to prove a correlation between a pre-operative parameter and surgical outcome; however, the authors did not comment on the need for surgical revision in patients with restenosis [6].

In 2016, Wormald and colleagues proposed DRAF III as the primary approach in patients with large supra agger frontal cells, pushing the drainage pathway of the frontal sinus medial, posterior or posteromedially, large supra bullar frontal cells, pushing the drainage pathway anteriorly, or frontal septal cells with a narrow frontal ostium (narrow anteroposterior diameter), pushing the drainage pathway laterally [3].

Despite endoscopic frontal sinus surgery having caught on in the last decade or so, to the best of our knowledge, no other attempts have yet been made at validating these measurements on a population of patients subjected to a DRAF III.

In addition, we feel that restenosis might not be the best outcome to measure surgical success after a DRAF III. As a matter of fact, the reported rates of surgical revision after restenosis go from 5 to 30%, according to study [27].

A shrinking of approximately 30% commonly occurs during the first 1 to 2 years after DRAF III because of wound healing alone, especially if mucosal stripping has occurred during surgery and bony surfaces have been left exposed. This might even be worsened by inappropriate post-operative care. However, it is not easy to determine which shrinking percentage becomes critical, i.e., causes symptoms’ recurrence, since it depends on the initial size of the intraoperative opening, and on the underlying disease (studies have shown that allergic fungal sinusitis, eosinophilic rhinosinusitis and mucoceles are impairing factors), among other factors. In addition, it has to be stressed that among patients experiencing the same degree of restenosis, some may not present symptoms recurrence [9].

In the present study, we observed that the FOAP max displayed a good correlation with the observed diameter after DRAF III completion, and, most importantly, it was significantly lower in patients requiring surgical revision. For this reason, this parameter may be employed to improve pre-operative patient selection: for instance, patients with a small FOAP max might benefit from the outside-in DRAF III technique [28], and/or the surgeon might choose to harvest a mucosal flap or to insert a stent at the end of surgery to prevent restenosis in this subset of patients [29].

Interestingly, the FOAP min, which in the original study by Zhang et al. displayed a better correlation with the post-operative neo-ostium contour area with respect to the FOAP max, did not correlate with the observed diameter nor was it significantly different between patients who required surgical revision and those who did not.

We hypothesize that the FOAP max might be a better predictor of post-operative frontal sinus anteroposterior diameter (OD), and consequently of surgical outcome, with respect to the FOAP min, as the latter does not take into account the anteroposterior thickness of the frontal beak, which can and should be drilled as part of a DRAF III procedure.

Finally, even though it is not statistically associated with recurrence, it is the authors’ opinion that an acute angle between the glabella and nasion on the sagittal plane may hinder frontal ostium endoscopic visualization and correct insertion of drills into the frontal ostium area. Further studies may be necessary to confirm this hypothesis.

This study has a number of limitations, the main ones being its retrospective nature and small sample size. It must also be considered that post-operative CT scans were not performed with standardized timing but according to need. This obviously influenced the maximum anteroposterior diameter of the frontal sinus neo-ostium in the post-operatory CT scan, which we termed the obtained diameter (OD), which is sensitive to different stages of neoosteogenesis. A final limitation relates to the heterogeneity of the sample under analysis, given the different nature of sinonasal pathologies, which may have affected the recidivism rate. A larger sample would also have allowed us to evaluate the influence of further parameters, for example the nasal polyp score (NPS) or SNOT-22 in rhinosinusitis with nasal polyposis, on the risk of restenosis. The latter could be the subject of future, more in-depth studies. Furthermore, the limited size of the study cohort does not allow to define a precise cut-off for the FOAP max, below which the DRAF III intervention is at high risk of restenosis. A larger population and further studies are necessary to define this cut-off, which would have been interesting because there are still no unanimous opinions on the matter in the literature [30,31].

## 5. Conclusions

DRAF III procedures are becoming the mainstay for the treatment of recurrent benign frontal sinus pathology, and indications for a primary DRAF III are also increasing. With improved anatomical knowledge and pre-operative imaging being more and more advanced, there are virtually no absolute contraindications to this procedure; however, it is also clear that inter-individual anatomical variability greatly influences not only the technical difficulty of the surgical procedure, but also the post-operative outcomes. We have observed that the pre-operative maximum frontal sinus outflow tract anteroposterior diameter (FOAP max), which is easily measured on pre-operative mid-sagittal CT, is lower in patients who experience recurrence requiring surgical revision. It is therefore the present authors’ opinion that the FOAP max might be a good predictor of DRAF III outcome, since it correlates well with the maximum obtainable anteroposterior diameter of the frontal neo-ostium after a DRAF III procedure, which we named the obtained diameter. This finding should guide surgeons to at least adopt additional methods to prevent restenosis, such as reconstruction flaps or absorbable stents, if not to defer patients to open approaches in cases with a small FOAP max.

## Figures and Tables

**Table 1:** Patients’ characteristics.

Mean Age at Surgery in Years (Range)	53.7 (18–79)	N(%) Patients
Sex	Male	18 (62.1)
	Female	11 (37.9)
Diagnosis	Mucocele	15 (51.7)
	CRSsNP	7 (24.1)
	CRSwNP	3 (10.3)
	CRSwNP + mucocele	1 (3.4)
	Inverted papilloma	1 (3.4)
	Fibrous dysplasia	1 (3.4)
	Osteoma	1 (3.4)
Number of previous nasosinus surgeries	1	10 (34.49)
	2	9 (31.03)
	0	7 (24.14)
	3	2 (6.9)
	5	1 (3.4)
Previous Surgery	FESS	8 (27.59)
	Open frontal sinusotomy	5 (17.24)
	Orbital decompression + FESS	3 (10.34)
	DRAF IIA	2 (6.9)
	Repair of CSF fistula via craniotomy	1 (3.4)
	Maxillofacial surgery for frontal bone fracture	1 (3.4)
	Maxillofacial surgery for cleft lip and palate	1 (3.4)
Comorbidities	High blood pressure	5 (17.24)
	Allergic asthma	3 (10.34)
	Glaucoma	2 (6.9)
	Diabetes mellitus	1 (3.4)

FESS = functional endonasal sinus surgery; CSF = cerebrospinal fluid; CRSsNP = chronic rhinosinusitis without nasal polyps; CRSwNP = chronic rhinosinusitis with nasal polyps.

**Table 2:** Recurrences requiring surgical revision.

Mean Time Surgery—Relapse in Months	40 (14–80)	N(%) Patients
Number of patients experiencing recurrence	6 (20.7%)	
Diagnosis	Mucocele	2 (33.3)
	CRSsNP	2 (33.3)
	CRSwNP + mucocele	1 (16.7)
	Fibrous dysplasia	1 (16.7)
Type of relapse treatment	DRAF III revision	4 (66.7)
	Open frontal sinusotomy with bicoronal approach	1 (16.7)
	Referred for pharmacological therapy	1 (16.7)

**Table 3:** Results. FOAP: frontal sinus outflow anteroposterior diameter; OD: obtained diameter; G-N angle: glabella-nasion angle; RP: relapse patients; NRP: non-relapse patients. * Results of Mann–Whitney test. ** Results of linear correlation analysis (r coefficient: Pearson correlation coefficient). #: statistically significant.

	Entire Study Cohort: Median, IQR, Range	RP: Median, IQR, Range	NRP: Median, IQR, Range	Results of Pairwise Comparison between RP and NRP (*p*-Value, 95% CI) *	Correlation with OD (*p*-Value, r Coefficient, 95% CI) **
FOAP max (mm)	14, 4, 7.67–19.16	14.68, 1.14, 13.99–15.80	15.87, 3.84, 8.54–22.63	0.04 #, −4.65 to −0.21	0.001 #, 0.7, 0.42 to 0.86
FOAP min (mm)	8, 3.49, 4.63–15.08	5.72, 3.37, 4.91–10.80	8.46, 3.31, 4.63–15.08	0.07, −3.97 to 0.45	0.45, 0.17, −0.26 to 0.54
OD (mm)	12, 2.06, 8.28–16.29	10.44, 1.67, 9.24–12.57	10.66, 5.26, 8.28–16.29	0.79, −3.05 to 0.10	/
G-N angle	129, 21.5, 101–147	136, 5.25, 127–142	120.5, 3.5, 101–147	0.11, −27.00 to 4.00	0.49, −0.19, −0.64 to 0.35

## Data Availability

The original contributions presented in the study are included in the article, further inquiries can be directed to the corresponding authors.

## Abbreviations

FOAP	frontal sinus outflow tract anteroposterior diameter
OD	obtained anteroposterior diameter
CRSwNP	chronic rhinosinusitis with nasal polyps
CRSsNP	chronic rhinosinusitis without nasal polyps

## References

[1] Muhonen E.G., Goshtasbi K., Papagiannopoulos P., Kuan E.C. (2020). Appropriate extent of surgery for aspirin-exacerbated respiratory disease. World J. Otorhinolaryngol. Head Neck Surg..

[2] Naidoo Y., Bassiouni A., Keen M., Wormald P.J. (2013). Risk factors and outcomes for primary, revision, and modified Lothrop (Draf III) frontal sinus surgery. Int. Forum Allergy Rhinol..

[3] Wormald P.J., Hoseman W., Callejas C., Weber R.K., Kennedy D.W., Citardi M.J., Senior B.A., Smith T.L., Hwang P.H., Orlandi R.R. (2016). The International Frontal Sinus Anatomy Classification (IFAC) and Classification of the Extent of Endoscopic Frontal Sinus Surgery (EFSS). Int. Forum Allergy Rhinol..

[4] Patron V., Roussel L.M., Moreau S., Hitier M. (2021). How to identify the anterior cribriform plate in the medial approach to the frontal sinus. Eur. Ann. Otorhinolaryngol. Head Neck Dis..

[5] Valdes C.J., Bogado M., Samaha M. (2014). Causes of failure in endoscopic frontal sinus surgery in chronic rhinosinusitis patients. Int. Forum Allergy Rhinol..

[6] Zhang X., Ye T., Huang Z., Huang Q., Xian J., Li J., Zhou B. (2018). Clinical Predictors of Frontal Ostium Restenosis After Draf 3 Procedure for Refractory Chronic Rhinosinusitis. Am. J. Rhinol. Allergy..

[7] DeConde A.S., Smith T.L. (2016). Outcomes After Frontal Sinus Surgery: An Evidence-Based Review. Otolaryngol. Clin. N. Am..

[8] Casiano R., Livingston J.A. (1998). Endoscopic Lothrop procedure: The University of Miami experience. Am. J. Rhinol..

[9] Weber R.K., Hosemann W. (2015). Comprehensive review on endonasal endoscopic sinus surgery. GMS Curr. Top. Otorhinolaryngol. Head Neck Surg..

[10] Bozzato A., Arens C., Linxweiler M., Bozzato V., Jecker P., Hilger G., Welkoborsky H.-J., Zenk J., Pillong L. (2022). Multicenter Observational Study to Evaluate the Diagnostic Value of Sonography in Patients with Chronic Rhinosinusitis. Diagnostics.

[11] Nathan K., Majhi S.K., Bhardwaj R., Gupta A., Ponnusamy S., Basu C., Kaushal A. (2021). The Role of Diagnostic Nasal Endoscopy and a Computed Tomography Scan (Nose and PNS) in the Assessment of Chronic Rhinosinusitis: A Comparative Evaluation of the Two Techniques. Sinusitis.

[12] Shih L.C., Patel V.S., Choby G.W., Nakayama T., Hwang P.H. (2018). Evolution of the endoscopic modified Lothrop procedure: A systematic review and meta-analysis. Laryngoscope.

[13] Papatsoutsos E., Kalyvas A., Drosos E., Neromyliotis E., Koutsarnakis C., Komaitis S., Chatzinakis V., Stranjalis G., Georgalas C. (2022). Defining the limits and indications of the Draf III endoscopic approach to the lateral frontal sinus and maximizing visualization and maneuverability: A cadaveric and radiological study. Eur. Arch. Otorhinolaryngol..

[14] Ting J., Wu A., Metson R. (2014). Frontal sinus drillout (modified Lothrop procedure): Long-term results in 204 patients. Laryngoscope.

[15] Bortoli V.T., Martins R.F., Negri K.C. (2017). Study of Anthropometric Measurements of the Anterior Ethmoidal Artery using Three-dimensional Scanning on 300 Patients. Int. Arch. Otorhinolaryngol..

[16] El-Anwar M.W., Khazbak A.O., Eldib D.B., Algazzar H.Y. (2021). Anterior Ethmoidal Artery: A Computed Tomography Analysis and New Classifications. J. Neurol. Surg. B Skull Base.

[17] Ohira S., Matsuura K., Matsui H., Nakamura M., Kamiyama K., Kajiwara R., Inoue A., Wada K. (2021). Anatomical Factors that Can Predict the Structure of Lamina Papyracea for Endoscopic Sinus Surgery. Laryngoscope.

[18] Hamour A.F., Kus L., Monteiro E., Scheffler P., Lee J., Vescan A. (2020). Radiological Anatomy of the Olfactory Fossa: Is Skull Base Anatomy Really Ever “Safe”?. J. Neurol. Surg. B Skull Base.

[19] Doubi A., Albathi A., Sukyte-Raube D., Castelnuovo P., Alfawwaz F., AlQahtani A. (2021). Location of the Sphenoid Sinus Ostium in Relation to Adjacent Anatomical Landmarks. Ear Nose Throat J..

[20] Raseman J., Guryildirim M., Beer-Furlan A., Jhaveri M., Tajudeen B.A., Byrne R.W., Batra P.S. (2020). Preoperative Computed Tomography Imaging of the Sphenoid Sinus: Striving Towards Safe Transsphenoidal Surgery. J. Neurol. Surg. B Skull Base.

[21] Ganesh V.L., Dharanipathy S., Pavana V., Kumar A., Sebastian L.J.D., Garg A. (2024). A computed tomography (CT)-based morphometric study of various skull base parameters and their anatomical relationships relevant to endoscopic endonasal skull base surgery. Surg. Neurol. Int..

[22] Papavasileiou G., Hajiioannou J., Kapsalaki E., Bizakis I., Fezoulidis I., Vassiou K. (2020). Vidian canal and sphenoid sinus: An MDCT and cadaveric study of useful landmarks in skull base surgery. Surg. Radiol. Anat..

[23] Gross C.W., Harrison S.E. (2001). The modified Lothrop procedure: Indications, results, and complications. Otolaryngol. Clin. N. Am..

[24] Farhat F.T., Figueroa R.E., Kountakis S.E. (2005). Anatomic Measurements for the Endoscopic Modified Lothrop Procedure. Am. J. Rhinol..

[25] Burkart C.M., Zimmer L.A. (2011). Endoscopic modified Lothrop procedure: A radiographic anatomic study. Laryngoscope.

[26] Gheriani H., Al-Salman R., Habib A. (2020). Frontal ostium grade (FOG): A new computer tomography grading system for endoscopic frontal sinus surgery. Otolaryngol. Head Neck Surg..

[27] Georgalas C., Hansen F., Videler W.J.M., Fokkens W.J. (2011). Long terms results of Draf type III (modified endoscopic Lothrop) frontal sinus drainage procedure in 122 patients: A single centre experience. Rhinology.

[28] Noller M., Fischer J.L., Gudis D.A., Riley C.A. (2022). The Draf III procedure: A review of indications and techniques. World J. Otorhinolaryngol. Head Neck Surg..

[29] Santarelli G.D., Han J.K. (2016). Evaluation of the PROPEL^®^ mini sinus implant for the treatment of frontal sinus disease. Expert. Opin. Drug Deliv..

[30] Schlosser R.J., Zachmann G., Harrison S., Gross C.W. (2002). The endoscopic modified Lothrop: Long-term follow-up on 44 patients. Am. J. Rhinol..

[31] Weber R. (2009). Endonasal frontal sinus surgery. Part 2: Frontal sinus drainage type III (median drainage), tips and tricks, postoperative care. HNO.

